# Health Risks Due to Co-Exposure to Noise and Respirable Crystalline Silica Among Workers in the Open-Pit Mining Industry—Results of a Preliminary Study

**DOI:** 10.3390/toxics12110781

**Published:** 2024-10-27

**Authors:** Iryna Myshchenko, Małgorzata Pawlaczyk-Luszczynska, Adam Dudarewicz, Alicja Bortkiewicz

**Affiliations:** 1 Laboratory of Occupational Health and Safety, Department of Mining, Faculty of Geoengineering, Mining and Geology, Wroclaw University of Science and Technology, 27 S. Wyspianskiego St., 50-370 Wroclaw, Poland; 2 Department of Vibroacoustic Hazards, Nofer Institute of Occupational Medicine, St. Teresa 8, 91-348 Lodz, Poland; malgorzata.pawlaczyk@imp.lodz.pl (M.P.-L.); adam.dudarewicz@imp.lodz.pl (A.D.); 3 Nofer Collegium, Nofer Institute of Occupational Medicine, St. Teresa 8, 91-348 Lodz, Poland; alicja.bortkiewicz@imp.lodz.pl

**Keywords:** occupational exposure, respirable crystalline silica, occupational noise, noise-induced hearing loss, risk assessment, combined exposure, open-pit mines

## Abstract

Occupational exposure to carcinogenic respirable crystalline silica and noise requires a deeper understanding and an assessment of the possible health risks caused by their combined action. Data on individual exposure to respirable crystalline silica (RCS) and occupational noise (ON) was collected among 44 open-pit miners. The study group was divided into two groups according to the job tasks performed. The individual exposure, exceeding of maximum admissible concentration/intensity, and predicted hearing thresholds (HTs) (according to ISO 1999:2013) were compared between the groups directly participating in the technological process (group 1; N = 23) and performing auxiliary, supervising, or laboratory activities (group 2; N = 21). All the analysed indices were significantly higher for group 1; therefore, the job category may predict ON and RCS exposure among open-pit miners. A statistically significant relationship (r_s_ = 0.66, *p* < 0.05) was found between the time-weighted average (TWA) 8 h RCS and individual daily noise exposure levels. Exposure to noise in the course of employment causes the risk of hearing impairment (mean HTs for 2, 3, and 4 kHz > 25 dB) up to 74% and 4.4% (in the case of groups 1 and 2, respectively). Further studies are needed before conclusions concerning the effects of co-exposure to ON and RCS on open-pit miners can be made.

## 1. Introduction

The mining industry is one of the most hazardous occupations worldwide, so safety demands for mining workers are continually increasing. Workers are exposed to dust and noise while extracting mineral resources. It happens due to the characteristics of the technological process and the use of powerful machinery and equipment, which are the sources of occupational noise. During blasting, excavation, transportation, and rock crushing, silica is broken down into fine particles and inhaled by workers. In recent years, there have been concerted efforts within the mining industry to reduce the health risks associated with silica dust and noise exposure. The US Commission of Inquiry into Mine Health and Safety has emphasised the need for better control measures to protect miners, highlighting the high rates of occupational lung diseases among mine workers. Federal compliance records have shown that workers at open-pit mines have some of the highest respirable dust and noise exposure rates in the metal/nonmetal mining industry [1].

Both respirable crystalline silica (RCS) and occupational noise (ON) can individually pose significant risks to workers. Therefore, their isolated actions are described well enough in the scientific literature. Currently, approximately 5 million workers in the European Union and 2.3 million in the US are exposed to respirable crystalline silica [2]. Based on epidemiological and experimental research results, the Working Group of the International Agency for Research on Cancer (IARC) classified RCS as Group 1 carcinogens for humans. This fact implies that no safe exposure level exists, necessitating exposure levels for workers to be kept as low as technically feasible. After inhalation, RCS turns into biologically active dust which deposits in the lungs. It not only increases the risk of developing serious silica-related diseases including chronic obstructive pulmonary disease (COPD), silicosis, and lung cancer but is also related to the development of autoimmune disorders and cardiovascular impairment [2,3,4,5,6,7]. According to Directive (EU) 2017/2398, the majority of European countries accepted the occupational exposure limit (OEL) of RCS at the level of 0.1 mg/m^3^ [8]. Nevertheless, it is lower in Austria, Bulgaria, Estonia, Finland, Germany, Netherlands, Norway, Portugal, Slovenia, Spain, and Switzerland (from 0.025 to 0.075 mg/m^3^) [9]. Under the OSHA standard, the OEL for RCS is 0.05 mg/m^3^ [10].

Exposure to occupational noise (ON) is the most common risk factor in the working environment in Europe, and noise-induced hearing loss (NIHL) caused by ON is the most common occupational disease worldwide. When the A-weighted daily noise exposure level (i.e., the A-weighted equivalent continuous sound pressure level normalised to a nominal 8-h working day, L_EX,8h_) exceeds 80–85 dBA, NIHL can develop among workers. Noise exposure can cause the constriction of the cochlear blood vessels, disturb cell energy metabolism, etc. [11]. NIHL is a gradual, irreversible condition caused by damage to sensitive inner ear hair cells responsible for sound signal transmission into the brain. Hair cells cannot regenerate after damage or destruction, leading to permanent hearing loss. Elevated occupational noise can also cause indirect effects including hypertension, ischemic heart disease, sleep disturbance, and annoyance as well as affect immune resistance. Since no treatment can yet reverse noise-related damage completely, the early prognosis of hearing loss and preventive strategies play a pivotal role [12].

In addition to ON and RCS, open-pit mining workers are often exposed to other occupational hazards such as vibration (hand-arm and whole-body), carbon monoxide, and carbon dioxide as well as to ototoxic agents. These factors may have synergetic or additive effects, particularly between ON and vibration, contributing to hearing loss. Moreover, exposure to solvents and carbon monoxide could further exacerbate ototoxic risks. While these factors are highly relevant in occupational health, the focus of this study was specifically on co-exposure to noise and RCS. Further research should explore the combined effects of these additional exposures to provide a more comprehensive understanding of the risks faced by workers.

Various regulations and guidelines aimed to protect workers from RCS and ON often address each hazard separately. At the same time, a list of occupations where workers are simultaneously exposed to both RCS and ON includes mining and quarrying, construction, ceramic industry, glass manufacturing, road construction, maintenance, etc. [13,14,15]. Studying the combined effect of silica dust particles and noise allows a better understanding of how these two factors potentially exacerbate each other’s harmful effects.

Therefore, the necessity of conducting a preliminary study can be substantiated by several key factors:

1. Exploratory data collection on combined exposure: While individual risks associated with noise and RCS exposure are well documented, there is limited understanding of their combined effects on worker health in open-pit mining. A preliminary study can gather initial data to assess the potential interactions between these two hazards, allowing to explore whether co-exposure amplifies health risks such as hearing loss and respiratory disease. Early findings can guide future, more targeted research by identifying the areas of greatest concern.
2. Understanding the scope of the problem: Open-pit mining presents a unique working environment where workers are consistently exposed to both noise and RCS. A preliminary study is essential for establishing the extent of this problem within this specific industry. This initial research can help determine the prevalence of co-exposure, the levels of noise and silica that workers encounter, and the demographic or job-specific characteristics that might influence susceptibility to health risks.
3. Establishing a baseline for further research: Conducting a preliminary study allows for the establishment of a baseline for future studies. It helps generate preliminary hypotheses regarding the health effects of co-exposure, which can then be tested in larger-scale, more detailed studies. Without this initial understanding, future research could lack focus or direction. Early findings from the preliminary study will offer valuable insights into the key variables, risk factors, and outcomes that require further investigation.
4. Identifying vulnerable worker groups: Our research can help identify which worker subgroups (e.g., based on job role) may be more vulnerable to health issues arising from co-exposure. This early identification of at-risk groups is critical for designing effective health interventions and for prioritising protective measures.
5. Guiding occupational health interventions: The findings from the preliminary study can inform early interventions to protect workers before larger studies are completed. Even if full causal relationships are not yet established, identifying trends or early indications of health risks can lead to the prompt implementation of protective measures, such as improved ventilation systems, noise control strategies, or enhanced personal protective equipment. These early interventions can help reduce immediate risks to worker health while more detailed studies are being planned.

The primary aim encompasses understanding how co-exposure to ON and RCS affects miners, predicting hearing thresholds, and identifying whether job roles can serve as indicators of higher risk. It also sets the stage for future studies to explore the combined health impacts more deeply.

Specific tasks of the article include assessing individual exposure levels to RCS and ON among open-pit miners, comparing the exposure levels between different job tasks in open-pit mining, investigating the relationship between exposure to RCS and noise, predicting the risk of hearing impairment due to noise exposure, and evaluating whether job category predicts exposure to ON and RCS to highlight the need for further research on the effects of co-exposure to ON and RCS.

## 2. Materials and Methods

Data on occupational exposure had been collected by the accredited Laboratory of Occupational Health and Safety due to the obligatory routine measurements of harmful physical and chemical hazards in open-pit mines in the Lower Silesia Region (Poland) in 2023. The retrospective analysis included only individual noise exposure levels and concentrations of RCS evaluated at typical workplaces and carried out on the same day. Applying that criterion allowed us to choose 44 workplace measurements of noise and RCS concentrations collected in eight open-pit mines. All the collected noise and dust samples were from male employees. The studied subjects were divided into two groups according to the work task performed. The workers directly participating in the stone processing (the extraction of stones, their delivery, and machine and manual processing) comprised group 1 (N = 23). The personnel who performed auxiliary, laboratory, and supervising activities were included in group 2 (N = 21).

To evaluate noise exposure, sound pressure levels (SPLs) were measured following recommendations PN-EN ISO 9612:2011 and PN-N-01307:1994 using the SVANTEK sound analyser type SVAN 979. Individual daily noise exposure level was determined according to the task-based measurement strategy [16,17]. Thus, A-weighted equivalent continuous (L_Aeq,Tm_), A-weighted maximum (L_Amax_), and C-weighted peak (L_Cpeak_) SPLs were measured while performing each task. Considering the duration of each task and the L_Aeq,Tm_ levels during their performance, each worker’s daily noise exposure level (L_EX,8h_) was calculated. The expanded uncertainty of noise measurements was less than 2 dB and was not added to the final exposure estimation. To evaluate individual exposure to RCS, air samples were taken in the breathing area by cyclones equipped with filters (designed to capture particles around 4.0 μm in diameter) using sampling pumps SKC. Air samples were collected for at least six working hours. The analysis of crystalline silica concentration was performed by infrared absorption spectrophotometry. The results of RCS concentration presented in the article corresponded to 8-h working day. Individual levels of exposure were compared to the limit values accepted in Poland (maximum admissible concentration (MAC) of RCS equal to 0.1 mg/m^3^; maximum admissible intensity (MAI) of noise normalised to a nominal 8-h working day of 85 dB; and admissible maximum A-weighted sound pressure level and C-weighted peak sound pressure level equal to 115 and 135 dB, respectively [18]. The degree of MAI exceeding was calculated according to the following:

(1) $$k_{LEX,8h}=10^{\left(L_{EX,8h}-85\right)/10}$$

(2) $$k_{LAmax}=10^{\left(L_{A\;max}-115\right)/20}$$

(3) $$k_{LCpeak}=10^{\left(L_{Cpeak}-135\right)/20}$$

The resulting degree of MAI exceeding was the maximum of the three values obtained using the above formulae. In turn, the degree of MAC exceeding was assessed by the following formula:

(4) $$k=\frac{C}{0.1}$$

where C—time-weighted average (TWA) 8-h RCS (mg/m^3^).

The risk of NIHL in the study population due to regular occupational noise exposure was evaluated according to the standard ISO 1999:2013 [12]. This standard assumes that the hearing threshold level related to noise and age combines the hearing threshold related to age and the noise-induced permanent threshold shift. According to the standard, the risk of hearing damage related to exposure to noise and age is measured by the percentage (percentile) of people whose hearing threshold exceeds the assumed limit value (e.g., 25 or 45 dB). In turn, the risk of hearing damage related only to noise exposure is defined as the difference between the percentage (percentile) of the exposed and non-exposed to noise population (but otherwise equivalent to the noise-exposed population) whose hearing threshold exceeds the assumed limit value. In this study, a highly screened ontologically normal population (database A) was taken into consideration as the reference non-noise-exposed population. The statistical distribution of hearing threshold levels in the latter population was calculated according to the procedure described in ISO 7029.

Generally, the NIHL distribution is determined based on such parameters as age, gender, noise exposure level, and the time of exposure in years. However, in this study, considering that all the participants were men, gender distribution was excluded from the analysis. Furthermore, the calculation of NIHL risk assumed that study subjects start work at the age of 20 years, perform the same job, and are exposed to noise continuously throughout their working lives, i.e., up to 60–65 years. The risk of hearing loss was determined as an average for 1, 2, and 3 kHz or 2, 3, and 4 kHz. The 1, 2, and 3 kHz frequencies are particularly important for speech understanding and the so-called social hearing ability. In turn, in the frequency range of 2, 3, and 4 kHz, hearing loss caused by noise becomes visible earliest [11].

Descriptive statistics parameters (mean, standard deviation, 10th, 50th, and 90th percentile) were determined for the collected data. The difference between the two groups in various variables (e.g., daily noise exposure level) was evaluated using Student’s *t*-test or non-parametric Mann–Whitney U test, where applicable. The relationships between the variables were analysed using Spearman’s rank correlation coefficient. The Statistica v. 9.1. (StatSoft Inc., Tulsa, OK, USA) software package was used for statistical analysis. All the tests were conducted with an assumed *p* < 0.05 significance level.

## 3. Results

**Figure 1:**
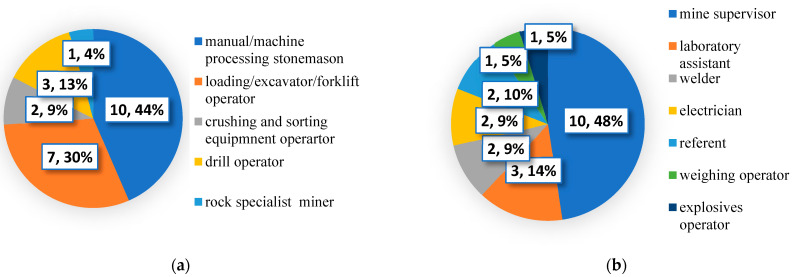
The number and percentage of subjects within group 1 (**a**) and group 2 (**b**) at the studied workplaces.

Rock and crushed stone products are usually broken by drilling and blasting and then are loaded into large haul tracks that deliver material to the processing operations. The last ones may include crushing, screening, size classification, handling, storage, etc. Therefore, chosen workplaces are typical for the open-pit mining industry and represent the whole cycle of technological processes (Figure 1).

**Figure 2:**
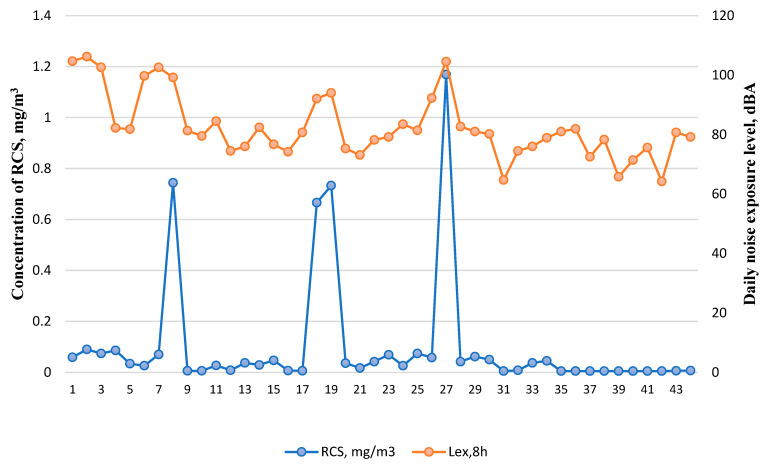
The concentrations of respirable crystalline silica and the noise exposure levels among the open-pit mining workers (N = 44).

The groups under study were characterised by different ON levels and RCS concentrations (Figure 2). The daily noise exposure level (L_EX,8h_) ranged from 64.2 to 106.2 dBA, while the RCS concentration varied from 0.005 to 1.169 mg/m^3^. The highest concentrations of RCS were found at the workplaces of the machine processing stonemasons (11.7- and 7.4 times exceeding MAC value) and manual processing stonemasons (7.5- and 6.7 times exceeding MAC values). The highest ON exposure level was measured at the workplaces of the drill operator (for example, 132-, 94- and 58-times MAI exceeding) and machine processing stonemason (89-times MAI exceeding).

Manual and machine processing stonemasons usually work in processing halls and such a high level of exposure to RCS is determined by the specific activities performed by the workers such as carving, cutting, shaping, smoothing, and polishing stones, etc. with the usage of manual or heavy machinery equipment. It is worth noticing that such working operations require physical activity and therefore lead to an increase in the blood flow and intensification of lung function, which may increase health risks due to more intensive inhaling of harmful dust particles. Workplace observations showed that dust extraction systems did not work properly mostly because dust flow direction changes rapidly depending on the needs of a technological process. A statistically significant relationship (Spearman’s rank correlation coefficient, r_s_ = 0.66 *p* < 0.05) was found between time-weighted average (TWA) 8-h RCS and individual daily noise exposure levels. Furthermore, significant differences were observed in occupational exposures between the groups of workers directly participating in the technological process (group 1) and performing auxiliary, laboratory, or supervising activities (group 2) (see Table 1).

**Figure 3:**
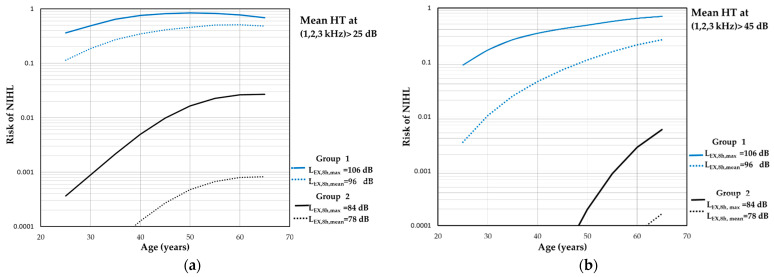
Results of the risk assessment of hearing impairment expressed as mean hearing threshold level at the frequencies of 1, 2, and 3 kHz > 25 dB (**a**) and >45 dB (**b**). The calculations are based on the energy mean and maximum values of daily noise exposure levels (i.e., L_EX,8h,mean_ and L_EX,8h,max_, respectively).

**Figure 4:**
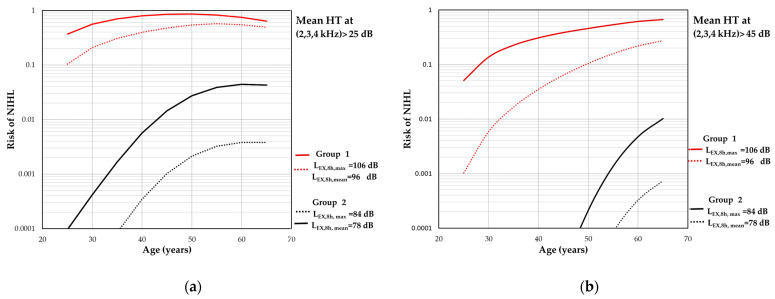
Results of the risk assessment of hearing impairment expressed as mean hearing threshold level at the frequencies of 2, 3, and 4 kHz > 25 dB (**a**) and > 45 dB (**b**). The calculations are based on the energy mean and maximum values of daily noise exposure levels (i.e., L_EX,8h,mean_ and L_EX,8h,max_, respectively).

According to the obtained results, the risk of NIHL is significantly higher in the group of workers directly participating in the technological process. As shown in Figure 3a and Figure 4a, depending on the length of employment (from 5 to 40 years which corresponds to age from 25 to 60 years), it fluctuates in the range of 11–51% for average hearing thresholds > 25 dB at 1, 2, and 3 kHz and 10–57% at 2, 3, and 4 kHz. The risk of NIHL at the maximal level of exposure for this group of employees ranged from 36 to 84% and 37 to 86% (for hearing thresholds > 25 dB at 1, 2, and 3 kHz and 2, 3, and 4 kHz, respectively). The highest risk was found for the workplace of drill operators (84% and 86% correspondingly at the age of 50) considering that the maximal levels of exposure during the whole period of working activity will be the same.

For the workers performing auxiliary, laboratory, or supervising activities, the risk of NIHL > 25 dB was estimated to be <<1% both for average hearing thresholds at 1, 2, and 3 kHz (Figure 3a) and at 2, 3, and 4 kHz (Figure 4a). In turn, after 40 years of employment, maximum daily noise exposure level can result in the risk of NIHL > 25 dB up to 2.67% and 4.4% (for 1, 2, and 3 and 2, 3, and 4 kHz, respectively).

As shown in Figure 3b and Figure 4b, permanent hearing threshold shifts due to noise exposure (expressed as mean at 1, 2, and 3 kHz and 2, 3, and 4 kHz) > 45 dB can be expected among up to 26% and 27% of the workers included in group 1. Exposure to maximal daily level of ON at 106 dB may result in hearing impairment (mean HTs > 45 dB at 1, 2, and 3 kHz) equal to 9–70% of the population and 5–67% of the workers (mean HTs > 45 dB at 2, 3, and 4 kHz). The maximum level of risk of noise-induced permanent threshold shifts > 45 dB for those frequencies did not exceed 1% of the working population participating in auxiliary, laboratory, or supervising activities at the open-pit mines.

## 4. Discussion

Mining activities are controlled according to the established national norms and regulations which consider the probability of health risks and potential danger of occupational hazards. According to the Regulation of the Minister of Health of Poland [19], workplace measurements of RCS (carcinogenic or mutagenic factor) shall be conducted: biannually—if its concentration was above 0.1 to 0.5 times the MAC value during the latest measurement; quarterly—if its concentration was above 0.5 times the MAC value during the latest measurement. In the case of occupational noise exposure, workplace measurements should be conducted at least once every two years, biennially—if during the last measurement, its intensity was above 0.2 to 0.5 times the MAI value; annually—if during the latest measurement, it was above 0.5 times the MAI value.

It is worth noting that occupational noise exposure levels can vary depending on specific tasks, work practices, and the effectiveness of control measures implemented by employers. For instance, during the drilling of concrete on construction sites, levels of RCS and noise increase with increasing bit wear. Therefore, contractors should replace bits that are worn based on usage history or bit tip wear patterns to reduce exposures [14]. Our study showed a correlation between ON and RCS exposure among open-pit mining workers which conforms with the literature data. For instance, research conducted among stone processing workers in Thailand revealed a statistically significant correlation between these occupational hazards. We also found a significant difference between groups of workers depending on the type of work performed. It supports the point of view that noise exposure level can be a predictable factor of respirable silica exposure in the stone-processing industry [20]. It is especially important because monitoring for silica requires expensive equipment, knowledgeable staff to perform the monitoring, and an available laboratory to perform the analysis. Similar conclusions have been drawn by Abbasi et al. [21].

Epidemiological studies have linked exposure to silica dust with serious lung diseases, such as silicosis and lung cancer. When workers are exposed to high levels of RCS, their lungs become scarred, leading to impaired breathing and other respiratory problems [3]. The probability of health risks caused by RCS largely depends on exposure duration, particulate size, and specific occupation activities. The first step in calculating the risk of cancer by occupational exposure to RCS is obtaining the level of cumulative silica exposure by multiplying the RCS concentration and years of exposure. The next step can be either the usage of data from epidemiological studies that rate specific levels of cumulative exposure to increased cancer risk or application based on the risk models that provide risk coefficients. The widely accepted and recommended model of Rice et al. establishes rate ratios for mortality from lung cancer of about 1.6 for the mean cumulative exposure to RCS compared with no exposure [4]. Their risk estimate predicts that 19 of every 1000 white male workers exposed to RCS at the level of 0.05 mg/m^3^ for their working lifetime would die from lung cancer related to silica. The predicted number of deaths in the same group exposed to 0.1 mg/m^3^ of RCS elevates nearly two times—up to 37/1000. This model did not take into consideration the smoking status or any other factors which can increase the risk of cancer. Another study estimated the excess lifetime risk of mortality from lung disease other than cancer (LDOC) and the onset of radiographic silicosis due to occupational exposure to RCS. The reanalysis of data from California diatomaceous earth workers revealed significant risks, with an excess lifetime risk for LDOC at 54 per 1000 and for silicosis at 75 per 1000, under the current OSHA permissible exposure limit [5]. These findings suggest that the existing occupational health standards for crystalline silica exposure permit substantially higher risks than the regulatory standards typically consider acceptable.

It was shown that the usage of more powerful cutting machines could change dust particle distributions and increase the relative abundance of RCS in respirable dust fraction [6]. The analysis of the occupational disease structure in Poland over the 2018–2020 years shows that the highest rate of pneumoconiosis incidence was recorded in the mining industry, and it was almost 10 times higher than in the next group of socioeconomic activities (i.e., education) and 18 times higher than the average in Poland. Despite the decline in employment in the mining industry, the incidence of pneumoconiosis in the same period increased by 49% [7]. The control of the emissions of dust particles containing RCS is quite a difficult task in open-pit mines. The factors affecting emissions include stone size distribution, the moisture content of the stones processed, the type of equipment, operating practices, and topographical and climatic factors (season, geographical location, and weather) [22]. In the case of manual or machine stone processing (where the highest concentrations of RCS were noted in this study), new fine particles are continually created by crushing, attrition, carving, and shaping stones. It leads to the reduction in moisture by evaporation.

Occupational noise exposure is amply studied [23]. It is well known that long-time exposure to noise can result in the apoptosis of hair cells and degeneration of spiral ganglion neurons. It continually decreases speech recognition, leads to increasing hearing thresholds and results in permanent hearing loss [24]. It is crucial for the mining and quarrying industry because this sector is atypical regarding age profile. According to Eurostat data [25], it has a significantly lower proportion of younger workers (only 12.9% of the employed are aged less than 30), whereas workers aged from 30 to 49 represent 63.2% of those employed within the industry. Recent studies linked ON exposure to diabetes [26], hypertension [27], and other cardiovascular effects [28], generally affecting human well-being, mental health, and life quality [29]. However, the traditional assessment of health risks based on a hierarchical representation of how events can develop is limited in situations of exposure to multiple occupational hazards, when the overall health effects may or may not be equal to the additive sum of each hazard alone. Cumulative effects may be synergistic, additive, or antagonistic.

Some studies state that exposure to both RCS and ON can result in a higher prevalence of health issues such as hearing loss, cardiovascular disease, and respiratory problems than exposure to either hazard alone. For instance, a study conducted by the U.S. National Institute for Occupational Safety and Health found that workers exposed to both silica dust and noise at the workplace had a significantly higher risk of developing hearing loss compared to those exposed to noise only [1]. Moreover, recent research has shown that construction workers exposed to RCS have not only a significant decline in lung functions, but a statistically higher hearing deterioration index compared to a control group [30]. It underlines the necessity to conduct pure-tone audiometry (which can determine the degree, type, and configuration of hearing loss) and hearing tests among open-pit mining workers to compare the actual hearing thresholds with theoretical predictions based on ISO 1999. It may also help to identify persons with so-called “hidden hearing loss” when having hearing thresholds within normal limits (>25 dB HL), people can have difficulties hearing in noisy environments. Finally, it helps to answer the question about the potential contribution of RCS in hearing pathology. Despite the limited evidence linking exposure to RCS with hearing loss, such interaction may be a key element of pathological processes that begin from a violation of the external breathing function caused by a dust load. It leads to the development of hypoxia, the impoverishment of blood oxygen saturation, and the occurrence of disturbances in the cardiovascular system. Reduced blood flow can decrease the auditory function under the influence of additional acoustic load. Certainly, this point of view needs further research.

An analysis of the confounding effects of noise and dust among 1092 Chinese workers showed a significantly higher risk of hypertension than the workers exposed to dust or noise alone [31]. The authors concluded that this risk increased with age, unhealthy behaviour, and work tenure and was more significant in men.

In a study of the combined effects of ON and dust among 6686 workers engaged in paper production in Sweden, Toren et al. [32] showed that high noise exposure levels (>90 dBA) and paper dust resulted in higher ischemic stroke mortality. In the context of paper production, the raw materials involved (such as wood pulp or recycled paper) typically do not contain significant amounts of crystalline silica. However, it is important to note that the processes within a paper mill or other industrial settings may involve equipment or materials that contain silica. For example, silica can be introduced through maintenance activities, the abrasion of certain machine components, or the use of additives in the papermaking process.

Therefore, the combined effect of RCS and ON should be studied at the level of (1) specific systems (auditory and respiratory); (2) integrated systems (cardiovascular); and (3) at the level of health indicators (subjective and objective). This approach will allow stratifying workers into different risk categories based on their degree of severity and, therefore, prioritise preventive measures for workers with more significant health risks.

The present study provides important insights into the combined occupational risks of respirable crystalline silica and noise exposure among open-pit miners. It focuses on dual exposure, an area that remains underexplored, especially in the mining industry. By examining real-world data and differentiating between job categories, the study highlights the significantly higher exposure levels faced by workers directly involved in the technological process. The identification of a statistically significant relationship between RCS and noise exposure (r_S_ = 0.66, *p* < 0.05) further underscores the importance of understanding these combined risks. Additionally, the quantification of hearing impairment risks provides valuable data for occupational health assessments and prevention strategies. Future research should aim to expand the sample size, incorporate longitudinal designs, and further investigate the health impacts of combined exposures, particularly the potential interaction between noise and RCS in the development of chronic conditions. These steps are crucial to developing comprehensive protective measures for workers in high-risk industries.

## Figures and Tables

**Table 1:** Summary results of the evaluation of exposure to ON and RCS in the studied groups. There were significant differences between groups 1 and 2 (*p* < 0.05).

Parameters	Group (N)		Total
	1 (23)	2 (21)	44
	M ± SE 10th/50th/90th percentile		
RCS, mg/m^3^	0.18 ± 0.064 0.0104/0.06/0.74	0.01 ± 0.003 0.005/0.006/0.037	0.10 ± 0.24 0.005/0.03/0.38
MAC exceedance	1.85 ± 0.64 0.104/0.6/7.39	0.14 ± 0.03 0.05/0.06/0.37	1.03 ± 0.38 0.05/0.32/3.87
A-weighted daily noise exposure level, dBA	88.7 ± 2.2 77.3/82.5/104.6	75.7 ± 1.2 64.9/46.0/82.3	82.5 ± 1.6 71.9/80.7/102.6
A-weighted maximum sound pressure level, dBA	99.1 ± 1.7 89.9/95.6/111.0	89.2 ± 1.9 74.2/87.6/104.9	94.8 ± 1.5 84.2/92.5/109.3
C-weighted peak sound pressure level, dBC	122.3 ± 1.67 107.7/125.0/131.1	109.9 ± 2.2 96.4/110.0/125.4	116.4 ± 1.7 101.5/118.9/130.4
MAI exceedance	22.2 ± 7.7 0.41/0.66/91.6	0.22 ± 0.04 0.024/0.13/0.54	11.7 ± 4.32 0.09/0.46/57.5

M—mean values; SE—standard error of mean values.

## Data Availability

The data will be made available upon request which should be sent to iryna.myshchenko@pwr.edu.pl and are subject to approval by all the named authors participating in this study/Supplementary Material.

## Supplementary Materials

The following supporting information can be downloaded at https://www.mdpi.com/article/10.3390/toxics12110781/s1, Table S1: Raw data on the levels of occupational noise, concentrations of respirable crystalline silica, and degrees of MAC/MAI exceeding among employees of open-pit mines.
